# Performance of two bone substitutes of novel cotton-like β-TCP/PDLGA and granular β-TCP on bone regeneration in the femoral bone defect of the Beagle dogs

**DOI:** 10.1016/j.bonr.2020.100718

**Published:** 2020-09-24

**Authors:** Yasuaki Okada, Yoshiaki Yamanaka, Kunitaka Menuki, Yukichi Zenke, Manabu Tsukamoto, Takafumi Tajima, Kenji Kosugi, Makoto Kawasaki, Eiichiro Nakamura, Naoka Toyota, Yasuhiro Kawabe, Akinori Sakai

**Affiliations:** a Department of Orthopaedic Surgery, School of Medicine, University of Occupational and Environmental Health, 1-1 Iseigaoka, Yahatanishi-ku, Kitakyushu 807-8555, Japan; b Department of Research and Development, TEIJIN MEDICAL TECHNOLOGIES Co., Ltd., 5322 Haga, Kita-ku, Okayama 701-1221, Japan

**Keywords:** PDLGA, β-TCP, Bone regeneration, Bone defect, Beagle dog

## Abstract

This study aimed to clarify whether novel cotton-like composite made of β-tricalcium phosphate (β-TCP) and poly(Dl-lactide-*co*-glycolide) (PDLGA) has a different effect on *in vivo* bone regeneration after bone defect than that of granular β-TCP. Five male Beagle dogs served as subjects. Cortical and medullary bone defect as non-through holes were made at the diaphysis of the bilateral femurs. One side was implanted with β-TCP/PDLGA (β-TCP/PDLGA group) and the other side was implanted with granular β-TCP (β-TCP group). At 4 weeks after implantation, we found no significant differences in the percentages of newly formed bone area and fibrous tissue area in the bone defect between the two groups. The β-TCP/PDLGA group showed more uniform filling on the surface and earlier disappearance of the material in the medullary region, and there were fewer inflammatory cells and osteoclasts in the bone defect in the β-TCP/PDLGA group. In conclusion, β-TCP/PDLGA performs better at filling the bone defect uniformly and disappears earlier at the cortical and medullary regions while causing less inflammation and bone resorption. Although bone formation activity of the β-TCP/PDLGA group in the cortical region was lower, the newly formed bone volume in bone defect of the β-TCP/PDLGA group was equal to that of the β-TCP group.

## Highlights

• Novel cotton-like composite made of β-TCP/PDLGA was compared with granular β-TCP.
• Cotton-like β-TCP/PDLGA performed better at filling the bone defect uniformly.
• Cotton-like β-TCP/PDLGA disappeared earlier at the implanted bone regions.
• β-TCP/PDLGA caused less inflammation and bone resorption in the bone defect.
• The newly formed bone volume in bone defect was equal between the two materials.

## 1 Introduction

Bone graft with autologous bone is the gold standard, but issues arise with surgical intervention at a healthy donor site, including hematoma, fracture, and pain. The harvesting of bone volume and structure is also limited. Thus, artificial alternatives to autologous bone for grafting are needed to fill the bone defects caused by trauma and curettage of benign bone tumors.

Biomaterials for bone substitutes must have biocompatibility with less immunogenicity and suitable biodegradability. Calcium phosphate compounds, such as hydroxyapatite and β-tricalcium phosphate (β-TCP), have been widely used as artificial bone in orthopaedic, maxillofacial, and plastic surgeries, because these materials show high biocompatibility and osteoconductivity (LeGeros, 2002). However, hydroxyapatite has a low degradation rate and takes a long time to be replaced by new bone tissue *in vivo* (Bucholz et al., 1989; Goto et al., 2001). Granular β-TCP is more commonly used, because of its biodegradability. The clinical medical needs of bone substitute include sufficient strength for supporting bone structure, free shape processing and plasticity, bone affinity, and promotion of bone formation in various clinical situations. In order to uniformly fill bone defects with complex shapes in a clinical setting, it is important to be able to freely shape the bone substitute without dropping granules (*i.e.*, materials like granular β-TCP) outside of the bone defect and to promote bone regeneration with earlier disappearance of artificial materials.

To meet these needs, we developed a cotton-like composite made of β-TCP, poly(DL-lactide-*co*-glycolide) (PDLGA), and gelatin. This material can be freely shaped and shows plasticity without breaking up or separating whereas the commercially available bone filler granular β–TCP has a fixed shape and is difficult for surgeons to pack uniformly into the bone defects with a complex shape. Moreover, earlier disappearance of this novel material is expected compared to granular β-TCP.

The purpose of this study is to clarify whether a novel cotton-like composite made of β-TCP and PDLGA has advantages over granular β-TCP with regard to *in vivo* bone regeneration after cortical and medullary bone defect.

## 2 Materials and methods

### 2.1 Materials

**Fig. 1:**
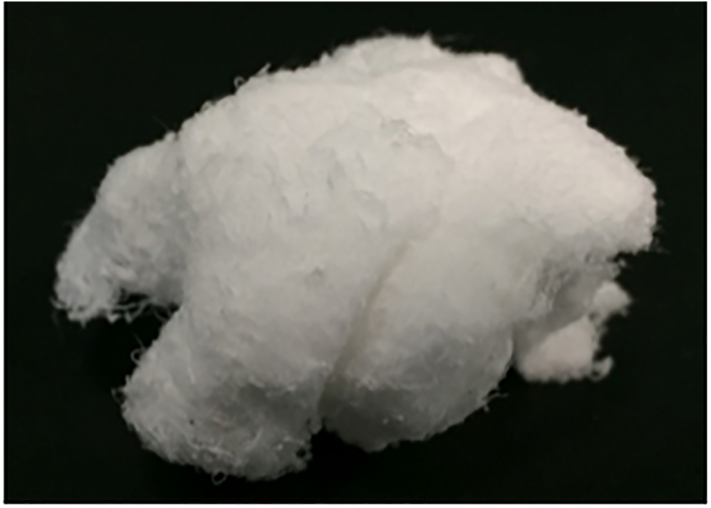
Novel bone substitute used for the study. Uncompressed β-TCP/PDLGA appears like cotton.

The novel cotton-like composite was made of β-TCP, PDLGA, and gelatin (Fig. 1). Copolymer of DLGA with a ratio (mol%) of DL-lactide to glycolic acid of 80:20 was dissolved in dichloromethane (FUJIFILM Wako Pure Chemical Corporation, Osaka, Japan). β-TCP (Taihei Chemical Industrial Co., Ltd., Osaka, Japan) was dispersed in the above solution (β-TCP/PDLGA 70:30 wt%). Cotton-like β-TCP/PDLGA was fabricated by spinning through blowout of gas and was sterilized by electron beam. This material was developed by TEIJIN MEDICAL TECHNOLOGIES Co., Ltd. (Osaka, Japan). Granular β-TCP, which is produced by Olympus Terumo Biomaterials Corp. (Tokyo, Japan), is commonly used in clinical settings.

### 2.2 Animal experiments

Experiments were carried out in the animal experimentation facility of Hamri Co., Ltd. (Ibaragi, Japan) and were approved by the Association of Assessment and Accreditation of Laboratory Animal Care International. Five healthy male Beagle dogs (Oriental Yeast Co., Ltd., Tokyo, Japan) aged 27 months old and with an approximate weight of 10 kg, were subjected to the experiment as described previously (Takigami et al., 2007). All animals were fed certified canine diet (DS-A; Oriental Yeast Co., Ltd.). Each dog was allowed free access to food and water and was housed in a hygienic stainless steel cage (88 cm wide, 92 cm long, 76 cm high, Shintoyo Manufactory Co., Ltd., Saitama, Japan) in an air-conditioned environment (temperature, 24 ± 4 °C, humidity, 50 ± 20%) that was illuminated from 07:00 to 19:00.

**Fig. 2:**
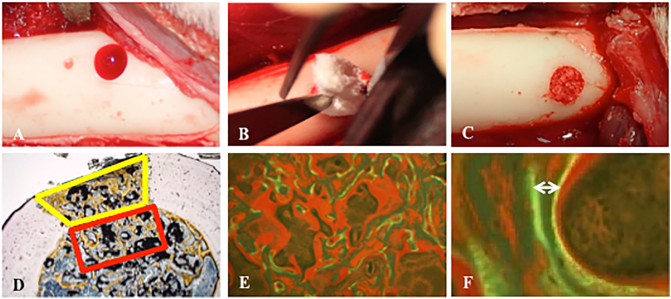
Surgical pictures and histological assessment. (A) drill-hole with 4 mm in diameter, (B) the defined amount of a novel cotton-like β-TCP/PDLGA material was implanted into the drill-hole, (C) drill-hole filled with β-TCP/PDLGA, (D) cortical region was measured at the area of cortical defect (yellow area) and medullary region was measured at the square area with 2 mm in width inside the cortical defect (red area), (E) formed bone was double-labeled with tetracycline and calcein, (F) inter-labels thickness was shown by two headed arrow.

All animals were anesthetized with induction using an intramuscular administration of 0.4 mL/kg of the mixture of ketamine hydrochloride and xylazine (1:1; v/v). They were intubated and ventilated under maintenance of anesthesia by inhalation of 1–2% isoflurane. After positioning in right-down and left-down lateral position, the bilateral thighs were prepared in a sterile manner. A 10-cm skin incision along the longitudinal femoral axis was made beginning just distal to the greater trochanter. Deep fascia was separated between the biceps femoris and the vastus lateralis and these muscles were separated. A cortical and medullary bone defect at the bilateral femurs, as non-through cylindrical holes with a 4-mm diameter and 7-mm depth, was made with an electric drill (Osada Electric Co., Ltd., Tokyo, Japan) after periosteal stripping at the diaphysis of the bilateral femurs (Fig. 2A). The bone defect on one side of the femur was implanted into the bone defect with 0.0321 g of β-TCP/PDLGA composite (β-TCP/PDLGA group; Fig. 2B) and that on the other side was implanted with 0.0719 g of granular β-TCP (β-TCP group), such that ~80% of porosity in the hole was filled with the respective materials. These graft materials were placed directly into the bone defects using forceps (Fig. 2C). Deep fascia and subcutaneous tissue were sutured and stainless steel staples were used for skin closure. Subjects received intramuscular administration of 0.02 mg/0.1 mL/kg of the painkiller buprenorphine and 5 mg/0.2 mL/kg of the antibacterial drug enrofloxacin once a day for 3 days. The dogs generally returned to full weight bearing by 2 days after surgical implantation.

Fluorescent bone labeling with subcutaneous injection of 20 mg/0.8 mL/kg of tetracycline (Sigma-Aldrich Japan, Tokyo, Japan) was performed 9 days before sacrifice and that of 10 mg/0.4 mL/kg of calcein (Dojindo Laboratories, Kumamoto, Japan) 2 days before sacrifice. At 4 weeks after surgical implantation, all animals were euthanized with death from exsanguination under thiamylal sodium anesthesia and then using a mixture of ketamine hydrochloride and xylazine. The bilateral femurs were harvested by removing the surrounding soft tissues. The bone samples at the region of interest were separated using a band saw. Specimens were fixed in 10% neutral buffered formalin solution overnight and then fixed in 70% ethyl alcohol.

The samples were assessed by micro-computed tomography (micro-CT) and histological examination. For histological assessment, they were embedded in paraffin after decalcification with 10% buffered EDTA for hematoxylin-eosin staining. They were also embedded in methyl methacrylate without decalcification for Villanueva Goldner bone staining.

**Fig. 3:**
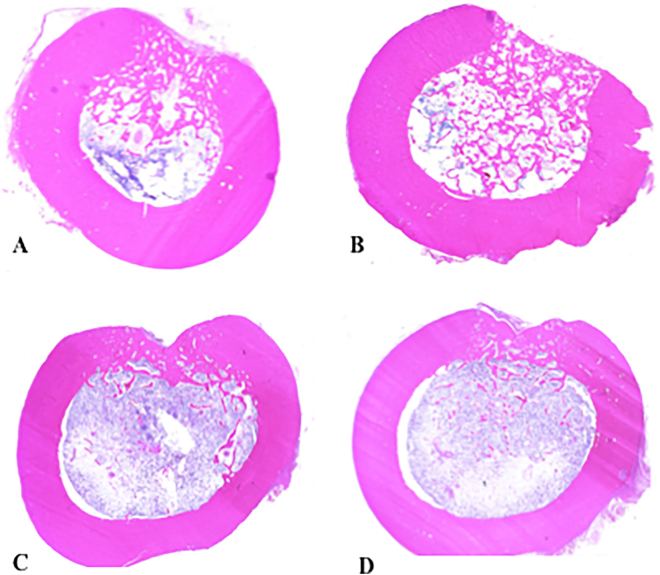
Cross sectional histological pictures stained with hematoxylin-eosin 4 and 12 weeks after implantation. (A) β-TCP/PDLGA and (B) β-TCP 4 weeks after implantation. (C) β-TCP/PDLGA and (D) β-TCP 12 weeks after implantation. Bone defects were almost completely repaired in the both groups 12 weeks after implantation without significant differences.

A preliminary experiment using two Beagle dogs was performed and the bone sample was harvested at 4 weeks and 12 weeks after implantation (Fig. 3A–D). At 12 weeks after implantation, the bone defect was completely repaired without marked difference between the β-TCP/PDLGA group and the β-TCP group. Thus, we determined that 4 weeks after implantation as appropriate time point for judgment of difference between these two materials.

All animal experimental protocols were approved in advance by the Ethics Committee for Animal Experimentation of Hamri Co., Ltd. (Authorization number; 16-H057). They were performed according to the Institutional Guidelines for Animal Experiments and Law No. 105 and Notification No. 6 of the Japanese Government. The investigation conformed to the Guide for the Care and Use of Laboratory Animals published by the US National Institutes of Health (NIH Publication No. 85-23, revised 1996).

### 2.3 Assessment

For micro-CT, the femoral embedded bone samples were analyzed by a micro-CT system (CosmoScan GX; Rigaku, Tokyo, Japan) at the following settings: 90 kVp, 88 μA, integration time 533.33 ms, field of view 25 mm, pixel size 50 μm.

For histological examination, the cortical region and the medullary region were determined for each cross-sectional specimen. The cortical region was the area of cortical defect between the cortical bones (Fig. 2D). The medullary region was the 2-mm-wide area inside the cortical defect. Fibrous tissue area was defined as area consisting of fibroblasts and collagen fiber bundles. Fibrous tissue area was measured in each region and expressed as the percentage of fibrous tissue area within each measured regional area. Measurement was performed at 40× magnification under a microscope (BX-43, Olympus Co., Tokyo, Japan) and shooting device (DP-22, Olympus Co.). The images were automatically processed through image alignment and tiling synthesis with the image processing software CellSence (Olympus) and image analytical software WinRoof ver.7.2 (Mitani Co., Fukui, Japan).

Angiogenesis was defined as capillaries observed at 400× magnification. The average number of capillaries measured at more than five high-powered fields (HPFs) was converted to a score according to the evaluation criteria of the International Organization for Standardization (10993-6, 2007-Biological evaluation of medical devices, Part 6, Tests for local effects after implantation, Annex E): 0 capillary/HPF = score 0, 1–3/HPF = score 1, 4–7/HPF = score 2, 8+/HPF = score 3. Inflammation was defined as respective inflammatory cells of polynuclear leukocytes, lymphocytes, and macrophages observed at 400× magnification. The respective average number of inflammatory cells measured at more than five HPFs was converted to a score in a similar manner: 0 inflammatory cell/HPF = score 0, 1–4/HPF = score 1, 5–9/HPF = score 2, 10+/HPF = score 3.

Bone histomorphometric analysis of non-decalcified specimens was performed as described previously (Tsukamoto et al., 2016). Non-decalcified 5-μm-thick coronal sections were cut on a microtome (model 2050 Supercut, Reichert-Jung, Heidelberg, Germany) and stained with Villanueva Goldner bone stain. Histomorphometry was performed using a semi-automatic image analysis system linked to a light microscope (Histometry-RT, System Supply, Nagano, Japan). Newly formed bone was measured in each region and expressed as the percentage of newly formed bone area in the measured regional area. Material residual area was measured in each region and expressed as the percentage of material residual area in the measured regional area. For the evaluation of new bone formation, we measured the inter-labels thickness, which is the distance between double labels stained with tetracycline and calcein viewed using fluorescence microscopy (Fig. 2E, F). The osteoid surface/bone surface (OS/BS: %) was determined. For bone resorption parameters, we also measured the osteoclast number/bone surface (N.Oc/BS: N/mm^2^) and the osteoclast surface/bone surface (Oc.S/BS: %) by identifying the cells that formed resorption lacunae on the trabecular bone surface and contained two or more nuclei as osteoclasts. The abbreviations for histomorphometric parameters were derived from the recommendations of the American Society for Bone and Mineral Research Histomorphometry Nomenclature Committee of the American Society for Bone and Mineral Research (Dempster et al., 2013).

For immunohistochemistry, bone samples were stained with osteocalcin to measure osteoblasts number in both the cortical and medullary regions. The osteocalcin-positive cells that exist at the bone surface were defined as osteoblasts. A 5-μm-thick section was cut from the paraffin blocks of the femoral specimens, and each section was mounted on a silane-coated glass slide. After deparaffinization, antigen retrieval was performed by immersing slides in proteinase K (Dako Code S3020, Agilent Technologies, Inc., Santa Clara, CA, USA) for 10 min, followed by soaking for 15 min at room temperature in 0.3% H_2_O_2_/methanol to block endogenous peroxidase. A serum-free protein block (Dako Code X0909, Agilent Technologies, Inc.) was performed for 20 min. Mouse anti-bovine osteocalcin monoclonal antibody (Clone OCG4, Code No. M044, Takara Bio Inc., Shiga, Japan), the primary antibody, was applied overnight at 4 °C. DAKO Envision+System-HRP labeled polymer anti-mouse antibody (DAKO Code K4001, Agilent Technologies, Inc.), the secondary antibody, was applied for 30 min at room temperature. The primary antibody was visualized using DAB/H_2_O_2_ solution (100–150 μL/slice) according to the manufacturer's instructions. The slide was counterstained with methyl green for 10 min.

### 2.4 Statistical analysis

Results were expressed as the mean ± standard error. The difference between the two groups was evaluated using Mann-Whitney *U* test. A *p* value of <0.05 was considered significant. The analysis was performed using Excel version 2011 (Microsoft, Redmond, WA, USA) and IBM SPSS Statistics software version 22.0 (IBM Japan, Tokyo, Japan).

## 3 Results

### 3.1 Macroscopic findings and micro-CT

**Fig. 4:**
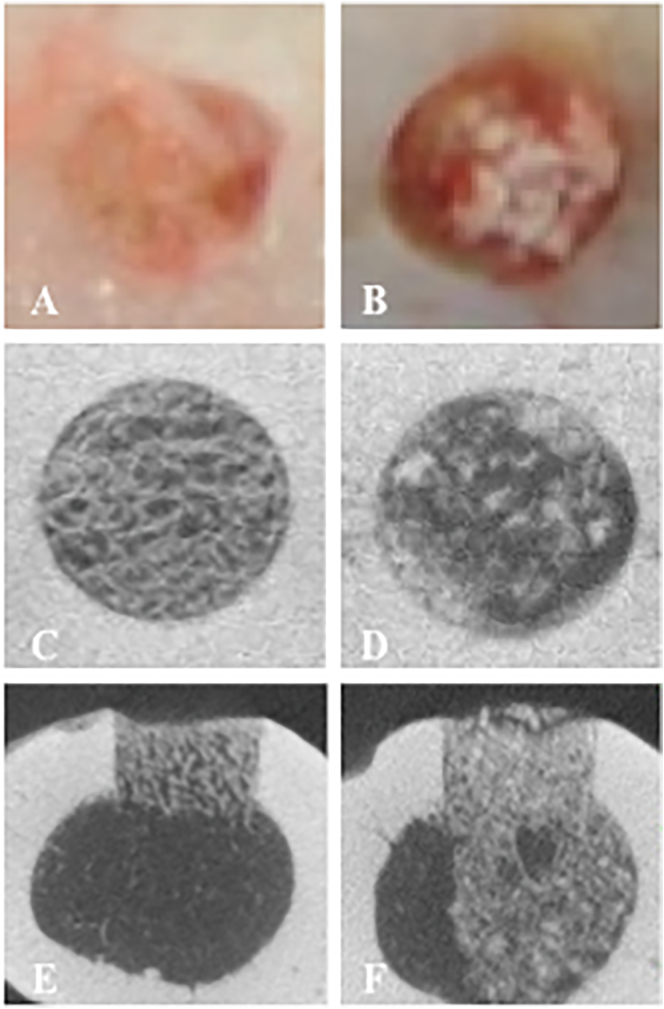
Microscopic and micro-CT pictures 4 weeks after implantation. Macroscopic observation on the surfaces of drill holes implanted with (A) β-TCP/PDLGA and (B) β-TCP. Micro-CT on the surface of drill holes implanted with (C) β-TCP/PDLGA and (D) β-TCP. Cross sectional micro-CT of drill holes implanted with (E) β-TCP/PDLGA and (F) β-TCP. Homogeneous surface was seen at (A) and (C), but remnants of β-TCP was seen at (B) and (D). Medullary cavity was remodeled at (E), but formed bone and material remnants were seen at medullary region of (F).

At the macroscopic level, the bone defects were filled with bony and fibrous tissues in both groups. More homogeneous filling on the surface of the bone defect was observed in the β-TCP/PDLGA group (Fig. 4A, B). Coronal views of micro-CT showed that the bone defect seemed to be filled more homogeneously in the β-TCP/PDLGA group compared to the β-TCP group (Fig. 4C, D). Axial views of micro-CT showed that the material disappeared in the medullary region in the β-TCP/PDLGA group, but not in the β-TCP group (Fig. 4E, F). The medullary cavity was remodeled in the β-TCP/PDLGA group.

### 3.2 Histomorphometry

**Fig. 5:**
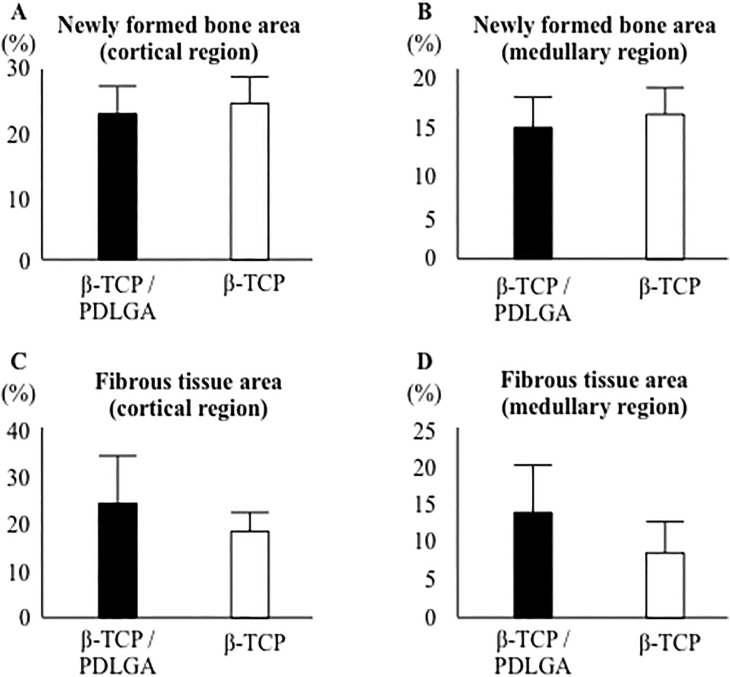
Percentages of newly formed bone area and fibrous tissue area at cortical and medullary regions. Newly formed bone area at (A) cortical region and (B) medullary region. Fibrous tissue area at (C) cortical region and (D) medullary region.

**Fig. 6:**
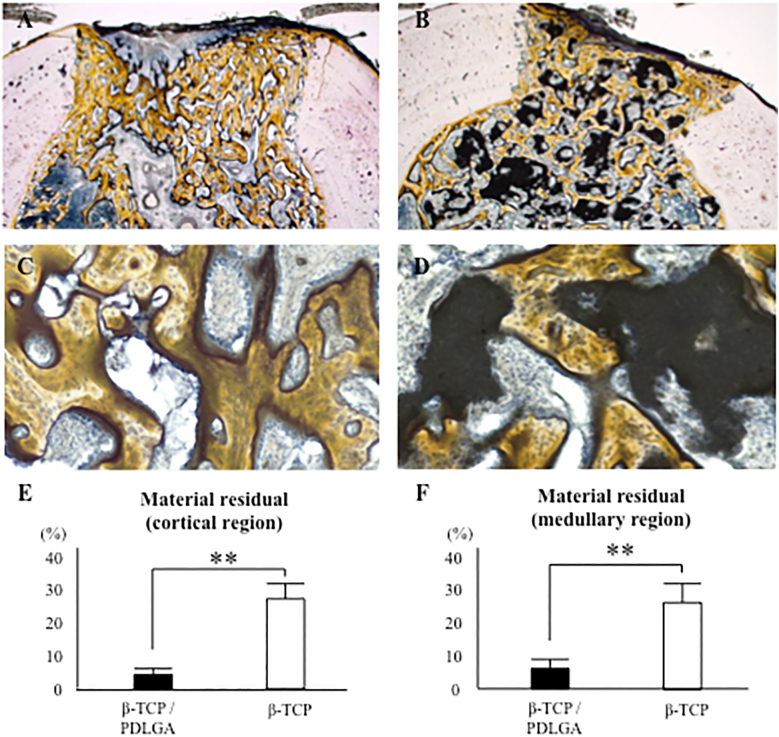
Cross sectional histological pictures stained with Villanueva Goldner bone stain 4 weeks after implantation. Low magnification pictures of (A) β-TCP/PDLGA and (B) β-TCP. High magnification pictures of (C) β-TCP/PDLGA and (D) β-TCP. Material residual appears black. Percentages of material residual at (E) cortical region and (F) medullary region. **: *p* < 0.01 by Mann-Whitney *U* test.

**Fig. 7:**
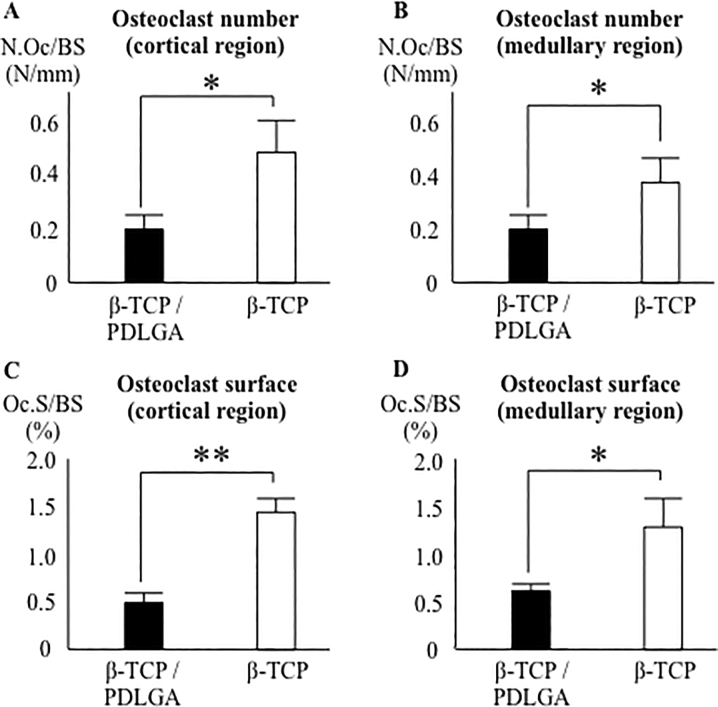
Osteoclast number and surface. Osteoclast number/bone surface (N.Oc/BS: N/mm) at (A) cortical region and (B) medullary region. Osteoclast surface/bone surface (Oc.S/BS: %) at (A) cortical region and (B) medullary region. *: *p* < 0.05, **: *p* < 0.01 by Mann-Whitney *U* test.

There were no significant differences in the percentages of newly formed bone area and fibrous tissue area between the two groups (Fig. 5A–D). The low and high magnification pictures of the β-TCP/PDLGA group showed less material in both the cortical and medullary regions compared to those of the β-TCP group (Fig. 6A–D). The residual rates of the materials were significantly lower in both the cortical and medullary regions of the β-TCP/PDLGA group compared to those of the β-TCP group (*p* < 0.01) (Fig. 6E, F). The values of osteoclast number (N.Oc/BS) were significantly lower in both the cortical and medullary regions of the β-TCP/PDLGA group compared to those of the β-TCP group (*p* < 0.05) (Fig. 7A, B). The values of osteoclast surface (Oc.S/BS) were significantly lower in the cortical region (*p* < 0.01) and in the medullary region (*p* < 0.05) of the β-TCP/PDLGA group compared to those of the β-TCP group (Fig. 7C, D).

### 3.3 Angiogenesis and inflammation

One to three capillaries per HPF at 400× magnification (score 1) were observed in all samples of the β-TCP/PDLGA group and the β-TCP group (Table 1). Concerning inflammatory cells, one to four polynuclear leukocytes per HPF at 400× magnification (score 1) were observed in two samples (40%) of the β-TCP/PDLGA group and four samples (80%) of the β-TCP group, and one to four lymphocytes per HPF at 400× magnification (score 1) were observed in two samples (40%) of the β-TCP/PDLGA group and three samples (60%) of the β-TCP group. No macrophages were observed in samples of the β-TCP/PDLGA group, but one to four macrophages per HPF at 400× magnification (score 1) were observed in three samples (60%) of the β-TCP group.

**Table 1:** Comparison of histological scores concerning angiogenesis and inflammation 4 weeks after implantation.

	Angiogenesis	Inflammation		
		Polynuclear leukocytes	Lymphocytes	Macrophages
β-TCP/PDLGA	1/1/1/1/1 (1)	0/0/1/0/1 (0.4)	0/1/1/0/0 (0.4)	0/0/0/0/0 (0)
β-TCP	1/1/1/1/1 (1)	1/1/1/0/1 (0.8)	1/1/1/0/0 (0.6)	1/0/1/0/1 (0.6)

Each value is score of the sample. Mean score of 5 samples in parenthesis. Score 1 indicates 1–3 capillaries/one high-powered field (HPF) for angiogenesis and 1–4 inflammatory cells such as polynuclear leukocytes, lymphocytes, and macrophages/HPF for inflammation at 400× magnification. Score 0 indicates no capillaries/HPF and no inflammatory cells/HPF. These scores were according to the evaluation criteria of the International Organization for Standardization (10993-6, 2007 Annex E).

### 3.4 Dynamic bone formation, osteoblasts, and osteoid

**Fig. 8:**
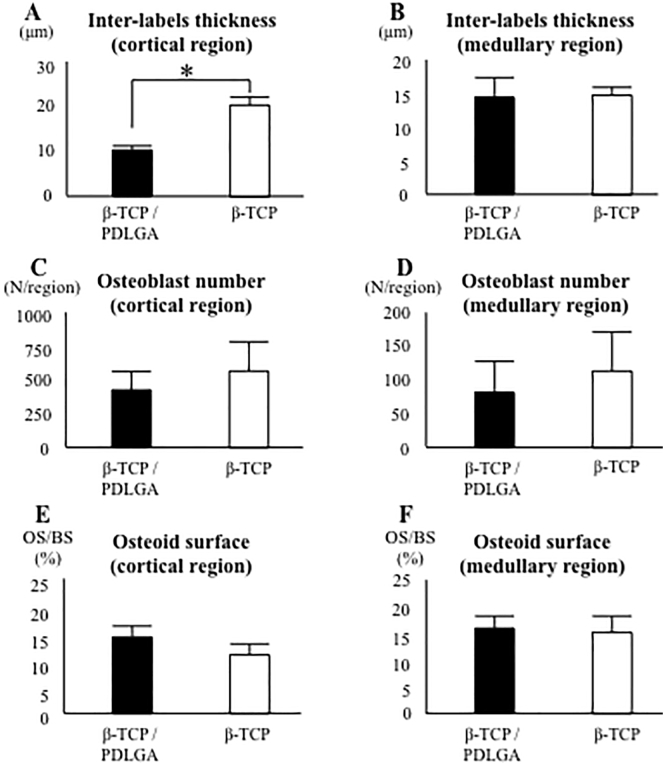
Bone formation ability, osteoblast number, and osteoid surface. Distance between double labels at (A) cortical region and (B) medullary regions. Osteoblast numbers at (C) cortical region and (D) medullary region. Osteoid surface/bone surface (OS/BS: %) at (E) cortical region and (F) medullary region. *: *p* < 0.05 by Mann-Whitney *U* test.

The distance between double labels in the cortical region of the β-TCP/PDLGA group was significantly shorter than that of the β-TCP group *(p* < 0.05), but not in the medullary region (Fig. 8A, B). There were no significant differences in the numbers of osteocalcin-positive osteoblasts in the cortical and medullary regions between the two groups (Fig. 8C, D). There were no significant differences in osteoid surface (OS/BS) in the cortical and medullary regions between the two groups (Fig. 8E, F).

## 4 Discussion

This study demonstrates that the novel cotton-like composite of β-TCP/PDLGA showed similar newly formed bone volume as the granular β-TCP in the bone defect, despite less bone formation activity in the cortical region, at 4 weeks after material implantation. Both groups showed equal number of osteocalcin-positive osteoblasts and equal surface of osteoid per bone surface. Use of the novel composite makes it easier to fill the bone defect uniformly, and the composite disappeared earlier at the cortical and medullary regions with less inflammatory cell infiltration and less osteoclastic bone resorption.

The inter-labels thickness of the β-TCP/PDLGA group in the cortical region was significantly shorter than that of the β-TCP group. However, the newly formed bone volume was equal between the two groups. The β-TCP/PDLGA had already disappeared and the medullary cavity was remodeled 4 weeks after implantation. The regeneration process seems to be earlier and more physiological in the β-TCP/PDLGA group compared to that in the β-TCP group.

The main absorption process of β-TCP was considered to be cell-mediated disintegration by tartrate-resistant acid phosphatase-positive cells (Tanaka et al., 2017). Osteoclast-mediated absorption of β-TCP is important for enabling bone formation. Material absorption was dependent upon the amount of implanted material and the type of bones (cortical or cancellous). β-TCP and poly-L-lactide (PLLA) respectively degrade to alkaline products and acidic products, leading to a neutral intraosseous environment (Zhu et al., 2013). In the current experiment, there were less material residual, less inflammatory cells, and less osteoclasts in the β-TCP/PDLGA group than those in the β-TCP group. Thus, we think that the reasons of earlier disappearance and physiological regeneration process would be implantation of less β-TCP (0.0321 g of the β-TCP/PDLGA group *versus* 0.0719 g of the β-TCP group) and neutralization of material degradation products.

Newly formed bone volume at the cortical region was equal in the two groups. The composite containing β-TCP was widely modified depending on usage. Tanaka et al. (2017) reported that an injectable complex of β-TCP granules and collagen supplemented with rhFGF-2 enabled cortical bone regeneration of rabbit tibiae. Ishida et al. (2019) reported that starfish-derived β-TCP had pores communicated to the inside of the material and showed improved cell proliferation compared with a commercially available β-TCP bone filler. By using our novel cotton-like composite made of β-TCP/PDLGA, it is easy to fill bone defects uniformly and evenly. However, this novel material does not have significant advantages over granular β-TCP bone filler with regard to new cortical bone formation. The development of other materials together with bone marrow aspiration or non-collagenous proteins may be needed to enhance bone formation (Sun et al., 2013).

In this study, we examined new bone formation under the condition of up to 80% porosity at the filled bone defect. β-TCP with 71–80% porosity (Chiba et al., 2016), block bone substitutes with three distinct ratios of hydroxyapatite: TCP with porosity >70% (Lim et al., 2015), and the scaffolds made of chitosan/hydroxyapatite/β-TCP (ratio of 50:30:20) with 70–80% porosity (Shavandi et al., 2016) were previously examined. An α-TCP scaffold coated with hybrid carbonate apatite/poly-epsilon-caprolactone (CO3Ap/PCL) maintained a fully interconnected structure with a porosity of 80–86% and achieved an improved compressive strength mimicking that of cancellous bone (Bang et al., 2015). Shavandi et al. (2015) compared two composites: chitosan/hydroxyapatite/β-TCP composites produced using squid pen-derived chitosan and commercial crab-derived chitosan. Thus, we think that porosity of a promising biomaterial for bone-tissue regeneration is approximately 80%.

Another cotton-like material was reported up to now (Schneider et al., 2009; Liu et al., 2016). The material was made of TCP and PLGA. Copolymer of LGA has a ratio (mol%) of lactide to glycolide of 85:15. Amorphous TCP nanoparticles and PLGA (TCP/PLGA 40:60 wt%) with a high mesh porosity of 95% were fabricated by low-temperature electrospinning. Thus, the copolymer ratio of lactide and glycolide, and the ratio of TCP and poly(lactide-*co*-glycolide) are different from our material used in this study. Schneider et al. (2009) reported that the new formed bone in PLGA and TCP/PLGA nanocomposite produced small holes and had a fine spongeous appearance. However, in our current experiment, both of the β-TCP/PDLGA group and the β-TCP group did not produce such small holes in the new formed bone as they reported (Schneider et al., 2009). The lack of small holes in the new formed bone in our material could be associated with less ratio of poly(lactide-co-glycolide) to TCP (β-TCP/PDLGA 70:30 wt%) compared to that in their material (TCP/PLGA 40:60 wt%).

The present study had several limitations. First, the bone was assessed at one time point after material implantation. It is unknown whether the peak value of inter-labels thickness, reflecting bone formation activity, appears before or after 4 weeks after material implantation. We do not have any data at the earlier times than 4 weeks. The time course of bone formation is not analyzed. Second, the quality of newly formed bone was unknown, as it is difficult to compare the strength of the newly formed bone itself between the two groups. Finally, the size and shape of the bone defect assessed is uniform. Although we would like to test the material in defects of various sizes and uneven complex shapes, it is quite difficult to set up adequate conditions to compare new bone formation between the two materials.

Despite these limitations, this was an efficacy study in which skilled veterinarians and orthopaedic surgeons treated dogs based on a defined porosity with novel and commercially available bone substitutes. The results of this study provide useful information and evidence for medical developers and innovators to achieve clinical success and sufficient improvement of bone substitutes. In conclusion, we confirmed that using a novel cotton-like composite of β-TCP and PDLGA makes it easier to fill the bone defect uniformly. This composite disappears earlier at the cortical and medullary regions while causing less inflammation. Although bone formation activity of the β-TCP/PDLGA group, as judged based on double labeling in the cortical region, is lower, the newly formed bone volume in bone defect of the β-TCP/PDLGA group was equal to that of the β-TCP group.

## Transparency document

Transparency document. Image 1

## Declaration of competing interest

The authors received financial support for the research from TEIJIN MEDICAL TECHNOLOGIES Co., Ltd. The study was jointly designed by the primary investigators and the sponsor. The sponsor was not involved in the analysis or interpretation of data.
